# The TLR3/IRF1/Type III IFN Axis Facilitates Antiviral Responses against Enterovirus Infections in the Intestine

**DOI:** 10.1128/mBio.02540-20

**Published:** 2020-11-17

**Authors:** Rui Su, Muhammad Adnan Shereen, Xiaofeng Zeng, Yicong Liang, Wen Li, Zhihui Ruan, Yongkui Li, Weiyong Liu, Yingle Liu, Kailang Wu, Zhen Luo, Jianguo Wu

**Affiliations:** a State Key Laboratory of Virology, College of Life Sciences, Wuhan University, Wuhan, China; b Guangdong Provincial Key Laboratory of Virology, Institute of Medical Microbiology, Jinan University, Guangzhou, China; c School of Forensic Medicine, Kunming Medical University, Kunming, China; Virginia Polytechnic Institute and State University

**Keywords:** enterovirus, type III interferons, intestine epithelial cells, Toll-like receptor 3, interferon regulatory factor 1, coxsackievirus B3, enterovirus 71, intestine, poliovirus 1, enterovirus infection, interferon-stimulated genes, neonatal C57BL/6J mice

## Abstract

Enteroviruses infect gastrointestinal epithelium cells, cause multiple human diseases, and present public health risks worldwide. However, the mechanisms underlying host immune responses in intestinal mucosa against the early enterovirus infections remain elusive. Here, we showed that human enteroviruses including enterovirus 71 (EV71), coxsackievirus B3 (CVB3), and poliovirus 1 (PV1) predominantly induce type III interferons (IFN-λ1 and IFN-λ2/3), rather than type I interferons (IFN-α and IFN-β), in cultured human normal and cancerous intestine epithelial cells (IECs), mouse intestine tissues, and human clinical intestine specimens. Mechanistic studies demonstrated that IFN-λ production is induced upon enterovirus infection through the Toll-like receptor 3/interferon regulatory factor 1 (TLR3/IRF1) signaling pathway in IECs. In turn, the supplementation of IFN-λ subsequently induces intrinsically antiviral responses against enterovirus replication. Notably, intraperitoneal injection in neonatal C57BL/6J mice with mouse recombinant IFN-λ2 protein represses EV71 replication and protects mice from viral lethal effects. Altogether, these results revealed a distinct mechanism by which the host elicited immune responses against enterovirus infections in intestine through activating the TLR3/IRF1/type III IFN axis. The new findings would provide an antiviral strategy for the prevention and treatment of enterovirus infections and associated diseases.

## Abstract (precis)

Enterovirus infections are significant sources of human diseases and public health risks worldwide, but little is known about the mechanism of innate immune response in host intestine epithelial surface during the viral replication. We reported the epithelial immune response in cultured human normal and cancerous cells (IECs), mouse tissues, and human clinical intestine specimens following infection with enterovirus 71. The results mechanistically revealed type III interferons (IFN-λ1 and IFN-λ2/3), rather than type I interferons (IFN-α and IFN-β), as the dominant production through TLR3/IRF1 signaling upon multiple human enterovirus infection, including enterovirus 71 (EV71), coxsackievirus B3 (CVB3), and poliovirus 1 (PV1). IFN-λ subsequently induced antiviral activity against enterovirus replication *in vitro* and *in vivo.* These studies uncovered the role of the novel process of type III IFN production involved in the TLR3/IRF1 pathway in host intestine upon enterovirus infection, which highlighted a regulatory manner of antiviral defense in intestine during enterovirus infection.

## INTRODUCTION

Enteroviruses are positive-sense single-stranded RNA viruses of the *Picornaviridae* family that include enteroviruses (EV), polioviruses (PV), coxsackieviruses (CV), and echoviruses (ECV) (1). Enterovirus infections cause human diseases ranging from mild to severe in outcomes, including encephalomyelitis, encephalitis, myocarditis, dilated cardiomyopathy, pleurodynia, acute flaccid paralysis, and even death (2–5). In mammals, the mucosal surfaces lining the gastrointestinal tracts have an important protective function (6). Enteroviruses enter the host through mucosal surfaces, and intestinal epithelial cells (IECs) are the first line of defense against invading pathogens (3). However, the defense mechanisms of gastrointestinal tract against enteroviral infections are not completely understood.

Interferons (IFNs) play a vital role in antiviral defense mediated by the innate immunity, and there are three classes of IFNs defined by their receptors. The type I IFN family comprises IFN-α and IFN-β that bind to IFNAR1/2 receptors to induce interferon-stimulated genes (ISGs). The type II IFN family is represented by a single member, IFN-γ, with affinity to IFN-γR1 and IFN-γR2 receptors. The type III IFN family is composed of four IFN-λ proteins in humans and two in mice, which function through a heterodimeric receptor complex, IFN-λR1 and IL-10R2 (7–10). Type I and type III IFNs have similar functions and transcriptional mechanisms involving interferon regulatory factors (IRF3 and IRF7), nuclear factor κB (NF-κB), and activator protein-1 (AP-1) (11, 12). However, differences exist in the mechanisms of expression and function of the two types of IFNs. Some mitogen-activated protein kinases (MAPKs) are selectively required for type I IFNs but not type III IFNs (13–16). While type I IFNs trigger ISG responses in several types of tissues, type III IFNs have prominent effects on tissues with mucosal surfaces (17). The antiviral activity of IFN-λ is more effective against viruses infecting epithelial cells of the respiratory tract (18, 19), gastrointestinal tract (20–22), and blood-brain barrier (BBB) (23).

As a typical member of the Toll-like receptor (TLR) family, TLR3 recognizes double-stranded RNA (dsRNA) formed during virus replication or released from necrotic cells (24, 25). TLR3 is an essential receptor and induced by enteroviruses in human IECs (26–28). IRF1, one of the IRF family of transcription factors, responds to viral infection (29, 30) and exerts antiviral activity in the early stages of viral infection in the respiratory tract (31). As the site of entry of enteroviruses, the intestinal tract is important for their replication and spread. Host responses in gastrointestinal epithelium may have a significant impact on enterovirus infection, but the mechanisms involved remain to be determined.

The present study demonstrated that EV71, CVB3, and PV1 predominantly induced IFN-λ1 and IFN-λ2/3 production in IECs through activating the TLR3/IRF1 signaling pathway. IFN-λ in turn stimulated an intrinsic antiviral action against enterovirus infections. Moreover, intraperitoneal injection of mouse recombinant IFN-λ2 protein resulted in the repression of EV71 infection in mice. Therefore, these results revealed a distinct mechanism by which the host elicits immune responses against enterovirus infections in the intestine through activating the TLR3/IRF1/type III IFN signaling pathway.

## RESULTS

### EV71 infection predominantly induces type III IFN production in human IECs.

**FIG 1:**
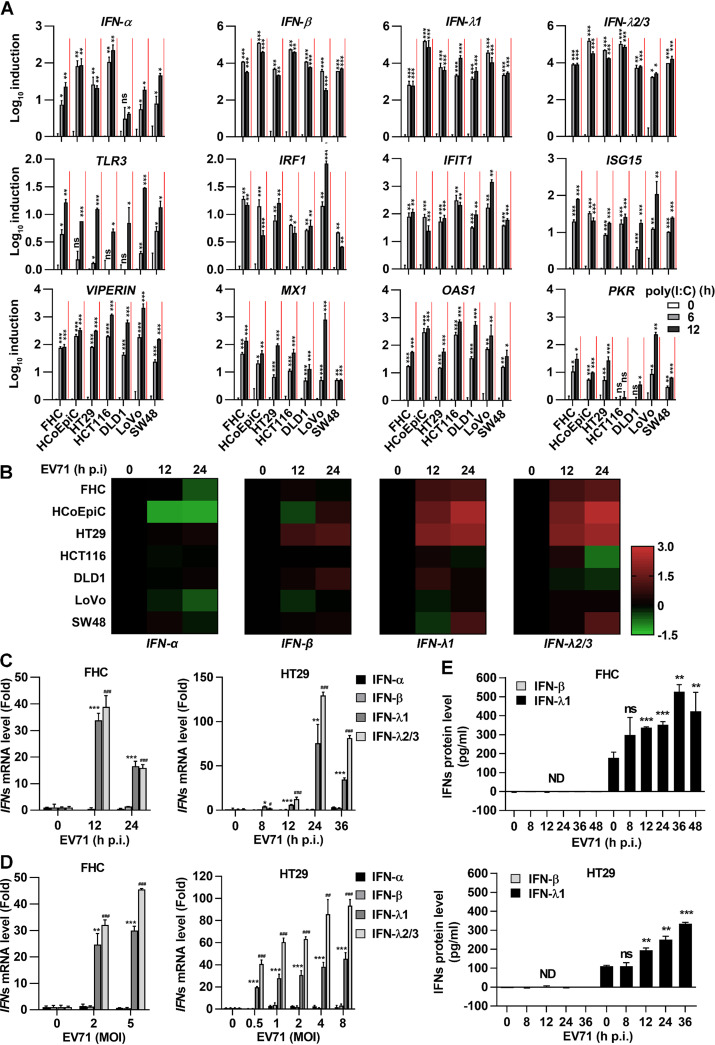
EV71 infection mainly induces type III interferon in IECs. (A) The human normal (FHC and HCoEpiC) and cancerous (HT29, HCT116, DLD1, LoVo, and SW48) intestinal cell lines were treated with poly(I·C) at a dose of 3 μg/ml for 6 h or 12 h. Human IFN and ISG gene expression was detected by qPCR analysis. Data are shown as a fold change (log_10_) relative to control (0-h group). (B) Seven human intestine cell lines were incubated with EV71 (MOI = 1) for 12 h or 24 h. The total mRNA of treated cells was extracted. Type I IFN (*IFN-α* [specific for *IFN-α1* and *IFN-α13*] and *IFN-β*) and type III IFN (*IFN-λ1* and *IFN-λ2/3*) mRNA levels were assessed by qPCR and visualized in a heatmap of expression values (log_10_ [fold change]). Data are representative of at least two independent experiments. (C and D) FHC cells were treated with EV71 at an MOI of 2 for 12 and 24 h (C) or at MOI of 2 and 5 for 12 h (D). HT29 cells were infected with EV71 at an MOI of 1 for 0, 8, 12, 24, and 36 h (C) or at MOI of 0, 0.5, 1, 2, 4, and 8 for 24 h (D). Type I IFN (*IFN-α* and *IFN-β*) and type III IFN (*IFN-λ1* and *IFN-λ2/3*) mRNA levels were detected by qPCR and normalized to the *GAPDH* mRNA level, and results are expressed as fold induction relative to control. * and # indicate the statistical significance for *IFN-λ1* and *IFN-λ2/3* mRNA levels relative to each control, respectively. (E) ELISA of IFN-β and IFN-λ1 production in the supernatant of FHC (MOI = 2) and HT29 (MOI = 1) cells treated with EV71 for the indicated time. Data are shown as mean ± SD and correspond to a representative experiment out of three performed. ns, nonsignificant; **, *P < *0.01; ***, *P < *0.001. ND, not detected. Statistical significance was determined by Student’s *t* test.

EV71 is a highly infectious RNA virus causing epidemics of hand, foot, and mouth disease (HFMD) and neurological diseases through early infection of host intestine (4, 32, 33). In this study, we attempted to investigate the effect of EV71 on the induction of IFN signaling in intestine epithelial cells (IECs). As some transformed cells with defects in IFN signaling and altered innate immune signaling have been reported (34), we initially selected and evaluated 7 human IEC lines including 2 human normal IEC lines (FHC and HCoEpiC) and 5 human cancerous IEC lines (HT29, HCT116, DLD1, LoVo, and SW48), which are suitable for enterovirus infection as described previously (34). The cells were treated with poly(I·C) to induce TLR3 signaling. The mRNAs of type I IFN, type III IFN, TLR3, IRF1, and ISGs (IFIT1, ISG15, VIPERIN, MX1, OAS1, and PKR) were induced at different levels upon the treatment with poly(I·C) in all 7 cell lines, especially in FHC, HCoEpiC, HT29, and LoVo cells (Fig. 1A), indicating that the TLR3 signaling pathway sustains the effect in these cells. Next, we evaluated the roles of EV71 infection in induction of the IFN signaling in the 7 cell lines. Upon EV71 infection, *IFN-α* mRNA was barely detected in all cells and *IFN-β* mRNA was slightly elevated in HT29 and DLD1 cells but not in other cells, whereas *IFN-λ1* and *IFN-λ2/3* mRNAs were significantly induced in HCoEpiC and HT29 cells and elevated in FHC and SW48 cells but not in DLD1 and LoVo cells (Fig. 1B), suggesting that type III IFNs are induced in human IECs. Furthermore, the effects of EV71 infection on induction of type I and type III IFNs were determined in FHC and HT29 cells. Infection of FHC and HT29 cells with EV71 triggered an obvious increase in *IFN-λ1* and *IFN-λ2/3* (type III IFNs) mRNAs, but no upregulation of *IFN-α* and *IFN-β* (type I IFNs) mRNAs (Fig. 1C and D). Moreover, compared to IFN-β, only IFN-λ1 protein was significantly induced in FHC and HT29 cells upon EV71 infection (Fig. 1E). EV71 replication as indicated by viral double-stranded RNA (dsRNA) (a replication intermediate) immunofluorescence (IF) analysis occurred at 12 h postinfection (p.i.) in FHC cells and from 8 h to 24 h postinfection in HT29 cells (see Fig. S1A in the supplemental material). The viral 3C protein was clearly detected in FHC and HT29 cells infected with EV71 (Fig. S1B and C). Collectively, these results demonstrated that EV71 infection predominantly induces type III IFN production in human IECs.

10.1128/mBio.02540-20.1 FIG S1 EV71 can replicate in human normal and cancerous IECs. (A) FHC and HT29 cells were infected with EV71 at an MOI of 2 and an MOI of 1 for indicated time, respectively, and stained with mouse anti-dsRNA (red) and DAPI (blue). The images were obtained from confocal microscopy. Bar = 20 μm. (B and C) FHC cells and HT29 cells were seeded in 6-well plates and infected with EV71 at an MOI of 2 and an MOI of 1 for indicated time, respectively (B). FHC cells (MOI = 0, 0.5, 1, 2, and 5 for 12 h) and HT29 cells (MOI = 0, 0.5, 1, 2, 4, and 8 for 24 h) were infected with EV71 (C). EV71 3C protein and GAPDH protein were detected by Western blotting. Download FIG S1, TIF file, 2.1 MB. Copyright © 2020 Su et al. 2020 Su et al. This content is distributed under the terms of the Creative Commons Attribution 4.0 International license.

### TLR3 mediates EV71-induced type III IFN production in human IECs.

**FIG 2:**
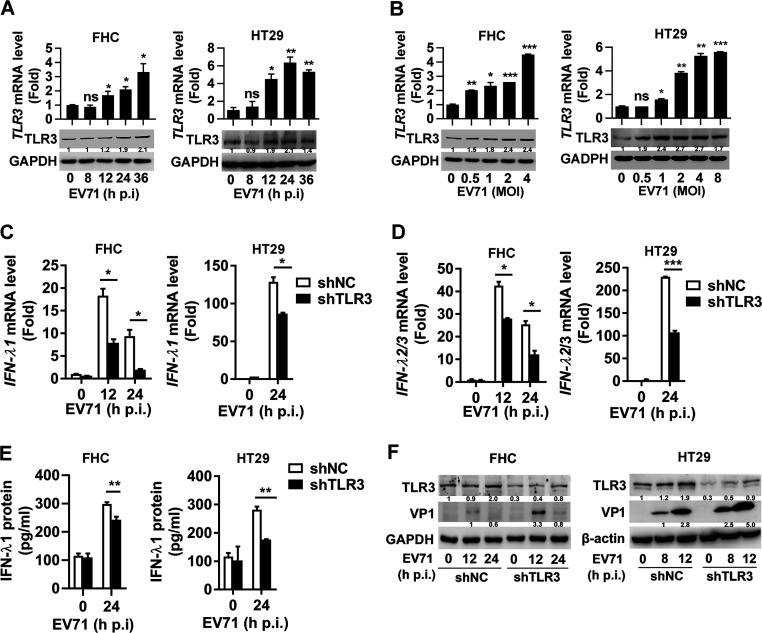
TLR3 is important for type III IFN production in human IECs upon EV71 infection. (A and B) EV71 infected FHC (MOI = 2) and HT29 (MOI = 1) cells for 8, 12, 24, or 36 h, respectively (A). FHC (MOI = 0, 0.5, 1, 2, and 4) and HT29 (MOI = 0, 0.5, 1, 2, 4, and 8) cells were treated with EV71 for 24 h (B). *TLR3* mRNA and TLR3 protein levels were measured by qPCR and Western blot analysis, respectively. (C and D) FHC cells stably expressing shNC or shTLR3 were infected with EV71 at an MOI of 2 for 12 or 24 h. HT29 cells stably expressing shNC or shTLR3 were infected with EV71 (MOI = 1) for 24 h. *IFN-λ1* (C) and *IFN-λ2/3* (D) mRNA levels were detected by qPCR. (E) FHC (MOI = 2) and HT29 (MOI = 1) cells stably expressing shNC or shTLR3 were treated with EV71 for 24 h. IFN-λ1 protein secreted in the cell supernatants was analyzed by ELISA. (F) EV71 infected FHC (0, 12, and 24 h, MOI = 2) and HT29 (0, 8, and 12 h, MOI = 1) shNC/shTLR3 stably expressing cells. TLR3, VP1, and GAPDH expressions were analyzed by Western blotting. Protein expression relative to internal control is quantified using Image J software. Data are shown as mean ± SD and correspond to a representative experiment out of three performed. ns, nonsignificant; *, *P < *0.05; **, *P < *0.01; ***, *P < *0.001. Statistical significance was determined by Student’s *t* test.

TLR3 is an essential receptor that initiates an antiviral response in human IECs upon rotavirus infections (27). We investigated the role of TLR3 in EV71-induced predominant production of type III IFN in IECs. The infection of FHC and HT29 cells with EV71 resulted in a robust increase in the levels of *TLR3* mRNA (Fig. 2A and B, top) and TLR3 protein (Fig. 2A and B, bottom), indicating that EV71-induced TLR3 may play a potential role in antiviral activity. The role of TLR3 in EV71-activated antiviral action was further evaluated by short-hairpin RNA (shRNA) targeting TLR3 (shTLR3), which efficiently attenuated TLR3 expression in HT29 cells (Fig. S2A). In comparison to the negative control (shNC), the *IFN-λ1* and *IFN-λ2/3* mRNA levels were induced by EV71 infection in FHC and HT29 cells, whereas such inductions were attenuated by shTLR3 (Fig. 2C and D). In parallel, EV71-mediated induction of IFN-λ1 protein was reduced by shTLR3 in FHC and HT29 cells (Fig. 2E). In addition, EV71 VP1 protein expression in FHC (Fig. 2F, left) and HT29 (Fig. 2F, right) cells along with viral titer in HT29 cells (Fig. S2B) was significantly enhanced by shTLR3, indicating that knockdown of TLR3 promotes EV71 replication. Therefore, TLR3 mediates EV71-induced production of type III IFNs in human IECs.

10.1128/mBio.02540-20.2 FIG S2 TLR3 and IRF1 play important roles in inhibition of EV71 replication. (A and C) HT29 cells stably expressing shNC and shTLR3, or shIRF1 were generated. The efficiencies of knockdown of TLR3 (A) or IRF1 (C) in HT29 cells were evaluated by Western blotting. (B and D) HT29 cells stably expressing shNC, shTLR3 cells (B), and shIRF1 cells (D) were infected with EV71 at an MOI of 1 for 0, 12, and 24 h. The virus titer in the culture supernatants was measured by TCID_50_ assay and expressed as TCID_50_/ml. Data are representative of at least three independent experiments and shown as mean ± SD. *, *P < *0.05; **, *P < *0.01. Statistical significance was determined by Student’s *t* test. Download FIG S2, TIF file, 0.8 MB. Copyright © 2020 Su et al. 2020 Su et al. This content is distributed under the terms of the Creative Commons Attribution 4.0 International license.

### IRF1 is important for TLR3-mediated type III IFN production upon EV71 infection.

**FIG 3:**
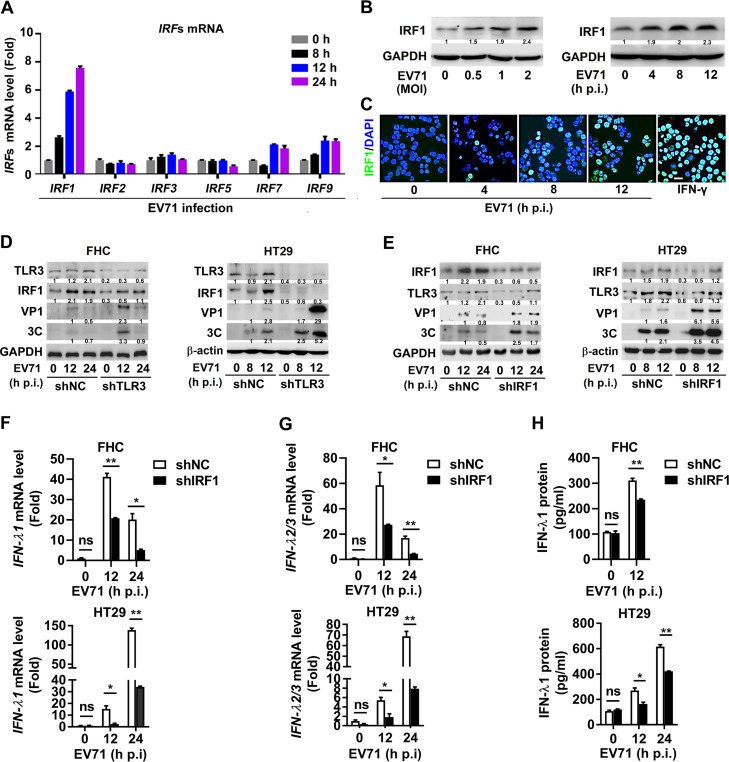
IRF1 is important for TLR3-mediated type III IFN production upon EV71 infection. (A) HT29 cells were infected with EV71 (MOI = 1) for the indicated time. Total RNA was extracted from the cells, and the level of *IRF1*, *IRF2*, *IRF3*, *IRF5*, *IRF7*, and *IRF9* mRNA was determined by qPCR. (B) HT29 cells were infected with EV71 at indicated MOI for 8 h or infected with EV71 at an MOI of 1 for 0, 4, 8, and 12 h. The cells were lysed, and IRF1 and GAPDH proteins were detected by Western blotting analyses. (C) HT29 cells were infected with EV71 at an MOI of 1 for 0, 4, 8, and 12 h or treated with 100 ng/ml of IFN-γ for 4 h, used as a positive control. The localization of IRF1 (green) and DAPI (blue) was analyzed by confocal microscopy. Bar = 20 μm. (D) FHC (MOI = 2) and HT29 (MOI = 1) stably expressing shNC or shTLR3 cells were infected with EV71 for the indicated time. Cell lysates were prepared, and the TLR3, IRF1, VP1, 3C, and GAPDH/β-actin proteins in the cell lysates were detected by Western blot analyses. (E to H) FHC and HT29 cells stably expressing shNC or shIRF1 were generated and then infected with EV71 at an MOI of 2 and an MOI of 1 for indicated times, respectively. Cell lysates were prepared, and the TLR3, IRF1, VP1, 3C, and GAPDH/β-actin proteins in the cell lysates were detected by Western blot analyses. The levels of *IFN-λ1* mRNA (F) and *IFN-λ2/3* mRNA (G) were detected by qPCR. The levels of IFN-λ1 protein secreted in the cell culture supernatants were determined by ELISA (H). All qPCR assays used *GAPDH* mRNA as an internal control. Results are expressed as fold induction relative to control. Protein expression relative to internal control is quantified using Image J software. Data are shown as mean ± SD and correspond to a representative experiment out of three performed. ns, nonsignificant; *, *P < *0.05; **, *P < *0.01. Statistical significance was determined by Student’s *t* test.

The distinct patterns of IRF-mediated induction of type I and III IFNs are linked to the differences in temporal and spatial responses to viral infection (16). Since TLR3 is required for the predominant production of type III IFNs in IECs infected with EV71, the effect of IRFs on the regulation of EV71 infection was explored. Initial data showed that infection of HT29 cells by EV71 resulted in a sharp increase in *IRF1* mRNA, modest induction of *IRF7* and *IRF9* mRNAs, and no effect on *IRF2*, *IRF3*, and *IRF5* mRNAs (Fig. 3A). Furthermore, the production of IRF1 protein was induced upon EV71 infection in a dose-dependent (Fig. 3B, left) and time-dependent (Fig. 3B, right) manner. Immunofluorescence analyses confirmed that the amount and nuclear location of IRF1 protein were continuously increased from 0 to 12 h postinfection (p.i.) in EV71-infected HT29 cells (Fig. 3C). Notably, EV71 promoted IRF1 production in the absence of shTLR3, whereas EV71-induced IRF1 production was attenuated in FHC and HT29 cells with shTLR3 (Fig. 3D), suggesting an essential function of TLR3 in EV71-induced IRF1 production in IECs.

The role of IRF1 in TLR3-mediated induction of type III IFN synthesis in EV71-infected IECs was also investigated using shRNA against IRF1 (shIRF1), which attenuated IRF1 expression in HT29 cells (Fig. S2C). Compared with the cells expressing shNC, EV71 VP1 and 3C protein expressions were significantly increased in FHC (Fig. 3E, left) and HT29 (Fig. 3E, right) cells along with the viral titer (Fig. S2D) in HT29 cells expressing shIRF1, indicating that knockdown of IRF1 leads to the facilitation of EV71 replication. Also, we observed that EV71 infection increased TLR3 protein level at 24 h p.i. in FHC cells and at 12 h p.i. in HT29 cells, whereas such production was attenuated in shIRF1-treated cells (Fig. 3E), suggesting that IRF1 is involved in EV71-induced TLR3 production. Collectively, these results indicated that there is a regulator*y* axis between TLR3 and IRF1 upon EV71 infection (Fig. 3D and E), which is consistent with IRF1-dependent TLR3 transcription as previously documented (30, 35). Moreover, the levels of *IFN-λ1* mRNA (Fig. 3F), *IFN-λ2/3* mRNA (Fig. 3G), and IFN-λ1 protein (Fig. 3H) were induced upon EV71 infection, whereas EV71-induced type III IFNs were reduced in shIRF1-treated FHC and HT29 cells. Taken together, IRF1 plays an important role in TLR3-mediated type III IFN production in IECs upon EV71 infection.

### Mouse intestine elicits antiviral responses during EV71 infection.

**FIG 4:**
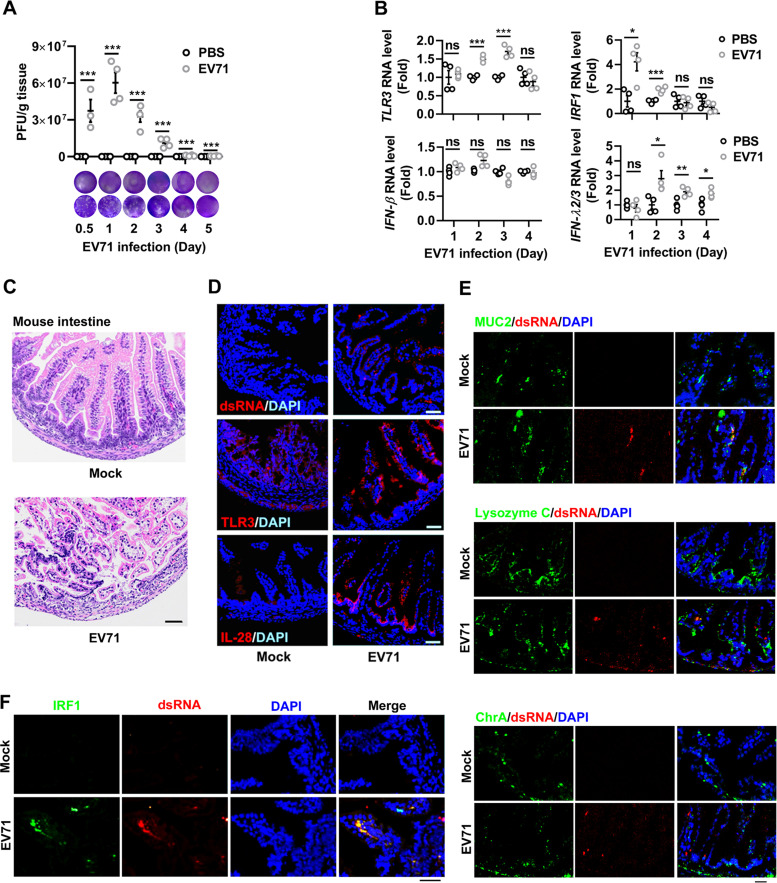
EV71 infection induces an antiviral response in mouse intestine. Three-day-old C57BL/6J mice were infected by intraperitoneal injection of 1 × 10^7^ PFU of EV71 (*n* = 20) or mock infected with PBS (*n* = 20). At 0.5, 1, 2, 3, 4, and 5 days after infection, animals were euthanized, and the small intestine tissues were collected. Each group, *n* = 3 to 4. (A) The EV71 titers of mice were quantified by plaque assay shown as PFU/g tissue. The representative images of viral plaque assay were displayed. (B) The levels of *TLR3*, *IRF1*, *IFN-β*, and *IFN-λ2/3* mRNAs were detected by qPCR. Data are shown as fold changes in RNA expression compared to the control group at 1 day to 4 days postinfection. Graphs represent the mean ± SEM. ns, nonsignificant; *, *P < *0.05; **, *P < *0.01; ***, *P < *0.001. Statistical significance was determined by Student’s *t* test. (C and D) Sections of the mouse small intestine at 3 days postinfection were stained by H&E (C) or fluorescently labeled using antibodies against TLR3, IL-28, or dsRNA (red) and DAPI to label the nuclei (blue) (D). (E) Immunofluorescence images from intestine were immunostained with DAPI (blue), intestine cell markers (MUC2, goblet cell marker; lysozyme C, Paneth cell marker; ChrA, enteroendocrine cell marker) (green), and dsRNA (red). (F) The small intestine sections of mice on day 3 postinfection from PBS and EV71 groups were immunostained with IRF1 (green), dsRNA (red), and DAPI (blue). All images are representative of each group (*n* = 3) with similar results. Bar = 100 μm.

Given that EV71 induced type III IFN through TLR3/IRF1 signaling in human IECs, the antiviral response in the small intestine of EV71-infected mice was analyzed *in vivo*. Previous studies demonstrated that intraperitoneal injection of EV71 in neonate mice leads to severe tissue damage and ultimately the death of the mice (36). On this basis, 3-day-old C57BL/6J mice were intraperitoneally injected with EV71. EV71 robustly replicated in the intestine tissue from day 0.5 to 1 of infection and then declined starting from day 2 to day 5 (Fig. 4A). During this interval, EV71 infection in mouse intestine upregulated *TLR3* mRNA on day 2 and day 3 (Fig. 4B) and *IRF1* mRNA on day 1 and day 2 (Fig. 4B), while it upregulated *IFN-λ2/3* mRNA on day 2 through day 4 (Fig. 4B). However, *IFN-β* mRNA was relatively unchanged from day 1 to day 4 postinfection (Fig. 4B). Histologically, epithelial damage and inflammatory cell infiltrations were developed in the EV71-infected mouse intestine (Fig. 4C). Immunofluorescence analyses showed expression of EV71 dsRNA (Fig. 4D, top, and Fig. S3A, left), accompanied by upregulated TLR3 (Fig. 4D, middle, and Fig. S3A, middle) and IL-28 (Fig. 4D, bottom, and Fig. S3A, right) in EV71-infected mouse intestine. We further assessed EV71 replication in intestines of mice, using a goblet cell marker (MUC2), a Paneth cell marker (lysozyme C), and an enteroendocrine marker (ChrA) with EV71 dsRNA. Immunofluorescence analysis revealed that viral dsRNA mainly localized with MUC2, suggesting that EV71 mostly infected goblet cells (Fig. 4E and Fig. S3B). Furthermore, IRF1 expression was significantly induced by the augmentation of viral RNA in the EV71-infected mouse intestine (Fig. 4F and Fig. S3C). Collectively, these results disclosed that EV71 infection predominantly induces type III IFN expression through the TLR3/IRF1/type III IFN regulator*y* axis in the immunopathogenesis and antiviral response to the viral infection in mouse intestine.

10.1128/mBio.02540-20.3 FIG S3 Immunofluorescence quantification analysis of antiviral responses in mice during EV71 infection. (A) Immunofluorescence analysis of viral dsRNA, TLR3, and IL28 expression in mouse small intestine treated with PBS or EV71. Expression was quantified by Image J software. (B and C) The colocalization between EV71 dsRNA and intestine markers (B) or IRF1 (C) was quantified by Image J software. All quantifications of images are representative of each group (*n* = 3) with similar results. Graphs show mean ± SD. *, *P < *0.05; **, *P < *0.01; ***, *P < *0.001. Statistical significance was determined by Student’s *t* test. Download FIG S3, TIF file, 0.8 MB. Copyright © 2020 Su et al. 2020 Su et al. This content is distributed under the terms of the Creative Commons Attribution 4.0 International license.

### EV71 infection is associated with the IRF1/TLR3/IFN-λ signaling activation in human intestine.

**FIG 5:**
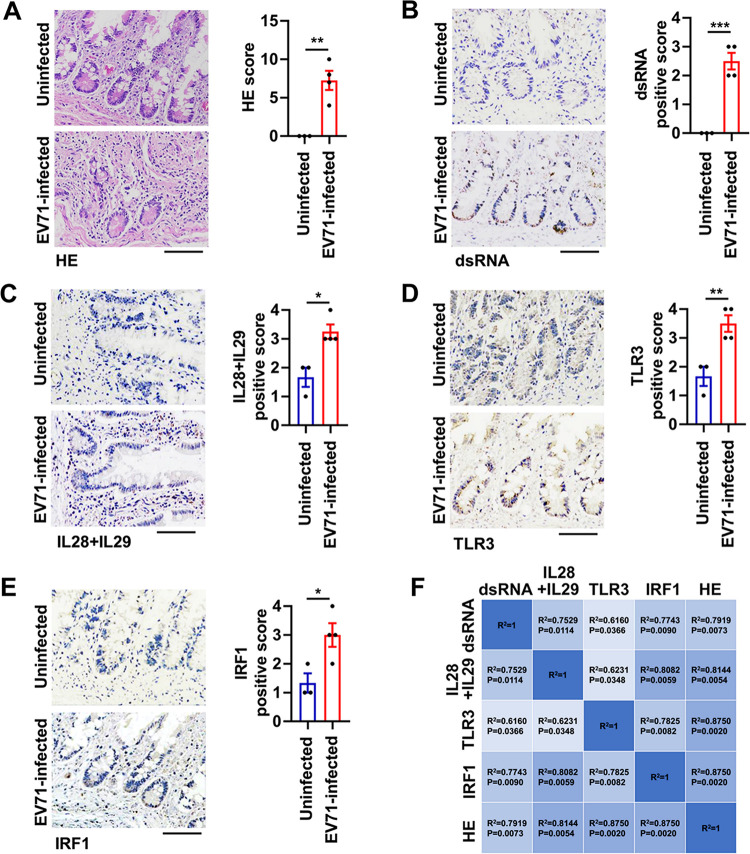
EV71 infection triggers the TLR3/IRF1/type III IFN signaling in human intestines. (A to E) Uninfected intestinal tissue samples (*n* = 3) and EV71-infected intestinal tissue specimens (*n* = 4) were collected from deceased human individuals. The small intestine tissue sections were analyzed by H&E staining (A) or immunohistochemistry using antibodies against dsRNA (B), IL-28+IL-29 proteins (C), TLR3 protein (D), and IRF1 protein (E). Positive staining is represented by brown coloration in these representative images. Light microscopy, bar = 100 μm. The relative levels of proteins were quantified with Image J software and are expressed as a positive score. Graphs show mean ± SEM. *, *P < *0.05; **, *P < *0.01; ***, *P < *0.001. Statistical significance was determined by Student’s *t* test. (F) Pearson’s correlation analyses of H&E scores, the viral dsRNA expression, IL-28+IL-29 production, TLR3 protein, and IRF1 protein in the 7 human intestine specimens. Statistical significance was determined by two-tailed *t* test.

To determine the role of TLR3, IRF1, and type III IFNs in EV71-induced pathogenesis in human intestine, histological analyses of seven specimens of the human intestine were performed (Table 1). Pathological changes including epithelial damage and inflammatory cell infiltrations were detected in the intestines of fatal cases of EV71 infection (Fig. 5A). The viral dsRNA was present in the intestine of EV71-infected patients (Fig. 5B). Additionally, histological analysis revealed that the expression of IL-28 and IL-29 (type III IFNs) (Fig. 5C), TLR3 (Fig. 5D), and IRF1 (Fig. 5E) was higher in the intestine of EV71-infected specimens than in that of uninfected ones, which is consistent with the observations in the intestines of EV71-infected mice. Moreover, correlation analyses demonstrated a highly significant positive correlation among the viral dsRNA, TLR3, IRF1, IL-28+IL-29, and hematoxylin and eosin (H&E) scores in human intestine specimens (Fig. 5F). Thus, human intestine may elicit an antiviral defense involving the TLR3/IRF1/type III IFN signaling pathway against EV71 infection.

**TABLE 1:** Demographic and baseline characteristics of EV71-negative individuals and EV71-positive patients^*a*^

Case no.	Age (yr)	Gender	EV71 infection
1	0.7	Male	−
2	2.9	Female	−
3	2.2	Male	−
4	1.3	Female	+
5	2.3	Male	+
6	3.0	Male	+
7	1.5	Female	+

a All EV71-positive patients were confirmed to be negative for other enteroviruses, were not suffering from any concurrent disease, and were negative for any serological marker indicative of autoimmune disease. EV71-negative individuals had no history of hand, foot, and mouth disease and served as negative controls. They were randomly selected but matched for sex and age.

### CVB3 and PV1 predominantly induce type III IFNs through the TLR3/IRF1 signaling in IECs.

**FIG 6:**
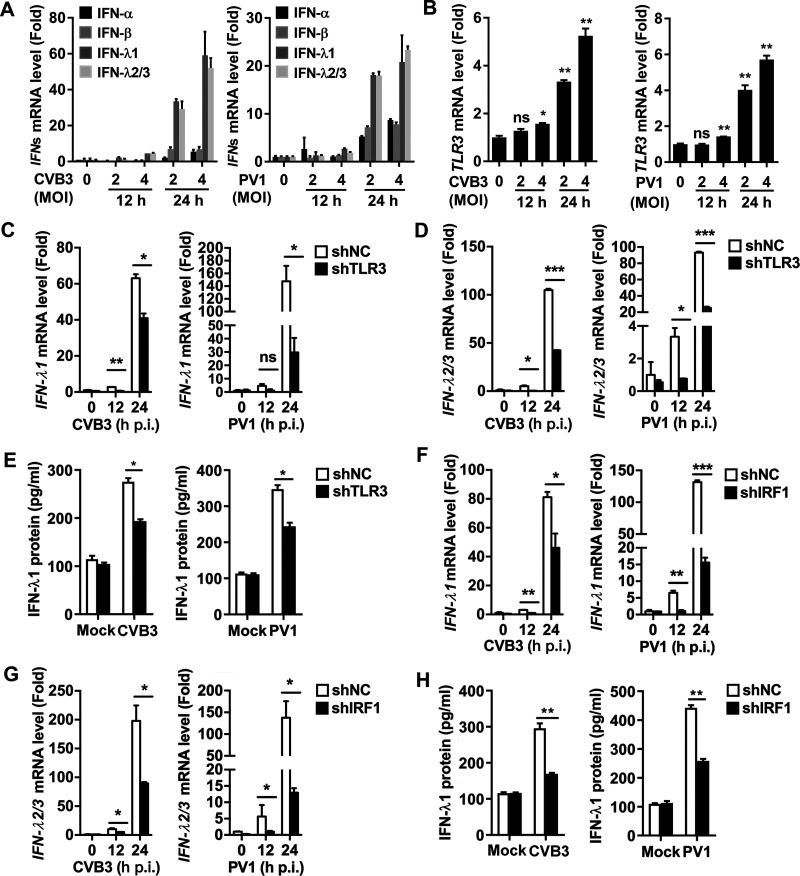
CVB3 and PV1 induce type III IFN production through the TLR3/IRF1 signaling in IECs. (A and B) HT29 cells were infected with CVB3 or PV1 at MOI of 0, 2, and 4 for 12 and 24 h. The levels of type I IFN (IFN-α and IFN-β) and type III IFN (IFN-λ1 and IFN-λ2/3) mRNAs (A) and TLR3 mRNA (B) were measured by qPCR. (C to E) HT29 cell lines stably expressing shNC or shTLR3 were infected with CVB3 and PV1 at an MOI of 2 for 12 h and 24 h. The levels of IFN-λ1 mRNA (C) and IFN-λ2/3 mRNA (D) were measured by qPCR. The levels of IFN-λ1 protein were determined by ELISA (E). (F to H) HT29 cell lines stably expressing shNC or shIRF1 were infected with CVB3 and PV1 at an MOI of 2 for 12 h and 24 h. The levels of IFN-λ1 mRNA (F) and IFN-λ2/3 mRNA (G) were measured by qPCR. The levels of IFN-λ1 protein were determined by ELISA (H). All qPCR assays used GAPDH mRNA as an internal control. Results are expressed as fold induction relative to control. Data are shown as mean ± SD and correspond to a representative experiment out of three performed. ns, nonsignificant; *, *P < *0.05; **, *P < *0.01; ***, *P < *0.001. Statistical significance was determined by Student’s *t* test.

EV71 induces type III IFN through activating the TLR3/IRF1 pathway, which raises the question of whether other enteroviruses also share the same mechanism leading to the triggering of antiviral defense in the intestine. We showed that in a manner similar to EV71, CVB3 and PV1 significantly induced the expression of *IFN-λ1* and *IFN-λ2/3* mRNA and slightly upregulated the expression of *IFN-α* and *IFN-β* mRNA in HT29 cells (Fig. 6A). In addition, *TLR3* mRNA was markedly promoted by CVB3 and PV1 infection (Fig. 6B). These results indicate that IFN-λ1, IFN-λ2/3, and TLR3 were predominantly activated in IECs upon the infections with enteroviruses. Next, the role of TLR3 in the regulation of type III IFN induction upon CVB3 and PV1 infections was investigated. The levels of *IFN-λ1* mRNA (Fig. 6C), *IFN-λ2/3* mRNA (Fig. 6D), and IFN-λ1 protein (Fig. 6E) were induced in HT29 cells upon CVB3 and PV1 infections, while the inductions of type III IFNs induced by CVB3 and PV1 were suppressed by shTLR3 (Fig. 6C to E). Moreover, the effect of IRF1 on the mediation of type III IFN induction upon CVB3 and PV1 infections was also evaluated. Similarly, the levels of *IFN-λ1* mRNA (Fig. 6F), *IFN-λ2/3* mRNA (Fig. 6G), and IFN-λ1 protein (Fig. 6H) were upregulated in HT29 cells upon the infections with CVB3 and PV1, while the productions of type III IFNs mediated by CVB3 and PV1 were attenuated by shIRF1 (Fig. 6F to H). Collectively, the predominant induction of type III IFNs through activating the TLR3/IRF1 pathway is a common characteristic of IECs upon the infections with enteroviruses.

### IFN-λ1 protein represses the replication of enteroviruses in IECs.

**FIG 7:**
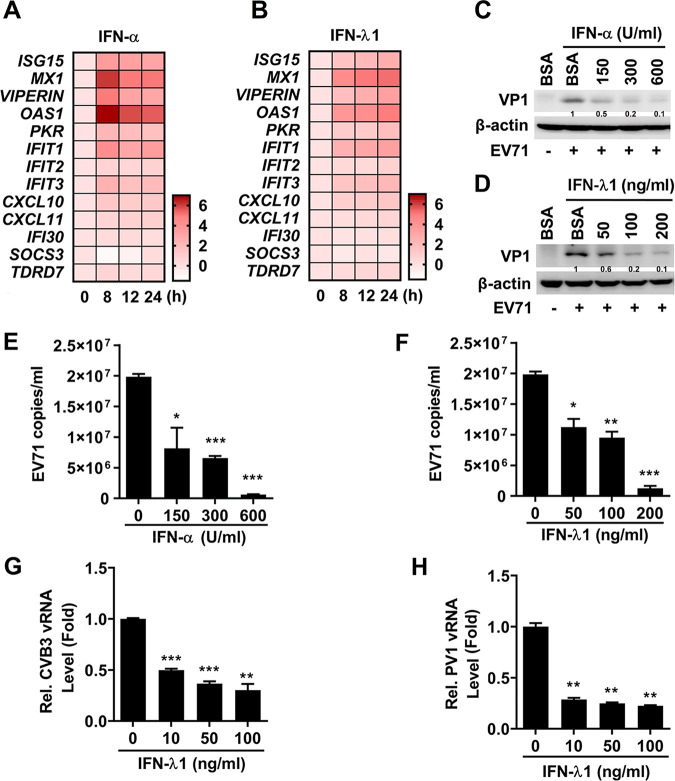
IFN-λ1 exerts antiviral activity against enterovirus replication in IECs. (A and B) HT29 cells were incubated with IFN-α (subtype IFN-α2b) (150 U/ml) (A) or IFN-λ1 (50 ng/ml) (B) for the indicated times. The mRNA levels of 13 IFN-inducible stimulated genes (ISGs) were assessed by qPCR. The data for all genes are normalized to GAPDH and visualized in a heatmap of expression values (log_2_ [fold change]). Data are representative of at least two independent experiments. (C to F) HT29 cells were plated in 12-well plates; treated with BSA, IFN-α (C and E), or IFN-λ1 (D and F) at different concentrations as indicated for 8 h; and infected with EV71 at an MOI of 1 for 16 h. The levels of EV71 VP1 protein and β-actin protein in the cell lysates were determined by Western blot analysis (C and D). EV71 copy numbers in the supernatants were determined by absolute qPCR analysis (E and F). (G and H) HT29 cells were seeded in 12-well plates, treated with IFN-λ1 at different concentrations for 24 h, and then infected with CVB3 (G) or infected with PV1 (H) at an MOI of 1 for 24 h. The levels of viral RNAs in the cell lysates were measured by qPCR. Protein expression relative to internal control is quantified using Image J software. Data are shown as mean ± SD and correspond to a representative experiment out of three. ns, nonsignificant; *, *P < *0.05; **, *P < *0.01; ***, *P < *0.001. Statistical significance was determined by Student’s *t* test.

As enteroviruses predominantly induce type III IFNs in IECs, the effect of type III IFNs on EV71 replication was assessed. Initially, HT29 cells were incubated with recombinant IFN-α protein or recombinant IFN-λ1 protein. In panel quantitative PCR (qPCR) analyses, expressions of 13 IFN-inducible antiviral genes (ISGs) indicated that similarly to IFN-α (Fig. 7A), expressions of several ISGs were also activated from 8 to 24 h after IFN-λ1 stimulation (Fig. 7B). Next, in agreement with a previous report (37), EV71 VP1 protein level (Fig. 7C and D) and the virus copy number (Fig. 7E and F) in HT29 cells were repressed by IFN-α protein (Fig. 7C and E), while they were restricted by IFN-λ1 protein in a dose-dependent fashion (Fig. 7D and F). Moreover, IFN-λ1 effectively repressed the replication of CVB3 (Fig. 7G) and PV1 (Fig. 7H) in HT29 cells. Altogether, type III IFN represses the replications of enteroviruses in IECs.

### Systemic treatment of mice with IFN-λ2 protein represses EV71 replication.

**FIG 8:**
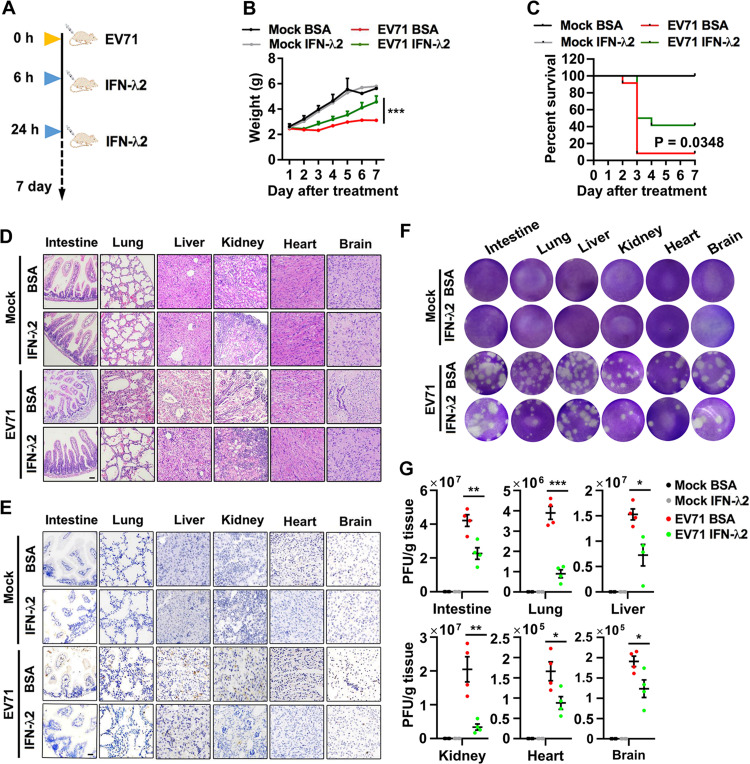
IFN-λ2 suppresses EV71 replication *in vivo*. (A) Schematic of mouse experiment. (B and C) Three-day-old suckling mice were injected with EV71 (intraperitoneally), 1 × 10^7^ PFU in 20 μl PBS, and then injected with IFN-λ2 (0.5 μg per mouse) at 6 and 24 h postinfection. The weight (B) and survival (C) were monitored for 7 days after infection (Mock, *n* = 6 in each group; EV71, *n* = 12 in each group). Statistical significance in weight and survival was calculated via two-way ANOVA and log rank tests, respectively. (D to G) Mouse tissues were stained with H&E (D) and IHC for viral dsRNA (E). Representative light microscopy images are shown. Bar = 100 μm. The EV71 titers in the intestine, lung, liver, kidney, heart, and brain tissues (each group, *n* = 4) at 3 days after the infection were determined by plaque assay (F) and quantified (G). Graphs show the mean ± SEM. *, *P < *0.05; **, *P < *0.01; ***, *P < *0.001. Statistical significance was determined by Student’s *t* test.

**FIG 9:**
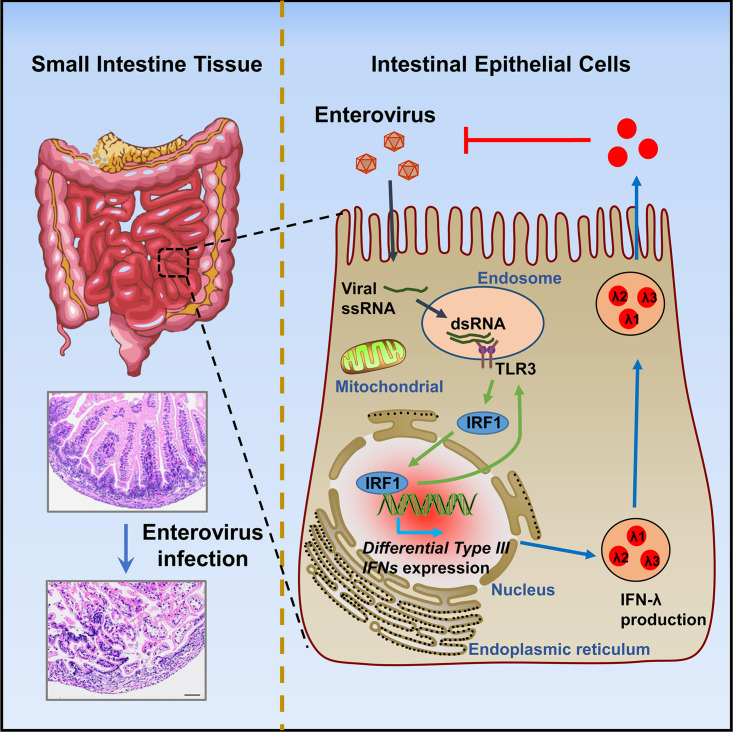
A proposed model underlying the TLR3/IRF1 signaling pathway. TLR3/IRF1 signaling regulates type III interferon production and antiviral activity in intestinal epithelial cells upon enterovirus infection. Upon infection by enterovirus in human or murine intestine tissues, intestinal epithelial cells are the first line of defense against virus infection. Viral dsRNA is recognized by TLR3 to trigger signaling events, and then TLR3-IRF1 signaling is activated. The IRF1 is translocated into the nucleus to induce the differential accumulation of type III interferon production, resulting in the activation of the innate immune antiviral response in intestinal epithelial cells.

The antiviral effect of type III IFN on EV71 replication was further examined in mice. It was previously documented that systemic treatment of suckling mice with IFN-λ represses rotavirus replication in the gut (26). Here, neonatal wild-type C57BL/6J mice were intraperitoneally injected with EV71 and subsequently treated with mouse recombinant IFN-λ2 protein or bovine serum albumin (BSA) (Fig. 8A). In comparison with EV71-infected mice treated with BSA, EV71-infected mice treated with IFN-λ2 displayed significantly less weight loss (Fig. 8B) and prolonged survival (Fig. 8C), suggesting that IFN-λ2 protects mice from the lethal effects of EV71 infection. H&E staining analyses showed that EV71-infected mice treated with BSA developed clear pathological changes, including epithelial damage and inflammatory cell infiltration in the intestine and lung, decreased hematopoiesis in the liver, atrophy of the kidney, severe necrotizing myocarditis in the heart, and neural injury in brain (Fig. 8D). All these alterations correspond to typical symptoms present in EV71-infected mice (38). Strikingly, the treatment with IFN-λ2 markedly alleviated the pathological changes in these tissues of infected mice (Fig. 8D). Immunohistochemistry (IHC) staining analyses indicated that in infected mice treated with BSA, the viral dsRNA was detected at a high level in the intestine; an intermediate level in the lung, liver, and kidney; and a low level in the heart and brain (Fig. 8E, top). However, in infected mice treated with IFN-λ2, the viral dsRNA was significantly decreased in all tissues (Fig. 8E, bottom). Similar results were obtained when the EV71 viral load in tissues was analyzed by plaque assay (Fig. 8F and G). Collectively, these results documented that systemic administration of IFN-λ2 protects mice against the effects of EV71 infection. Taken together, these results revealed a mechanism by which host intestine elicits immune responses against enterovirus infections through activating the TLR3/IRF1/type III IFN signaling pathway (Fig. 9).

## DISCUSSION

Enteroviruses invade the host through the gastrointestinal tract, and the intestinal epithelial cells sense invading viruses to elicit immune responses (39, 40). Here, we described an underlying mechanism of antiviral host defense involved in type III interferon in intestine epithelial cells upon enterovirus infection. Three-dimensional culture organoid emerged as a significant model to study enterovirus infection in intestine (41–44), with the requirements of containing all epithelial cell types of the normal gastrointestinal tract, retaining segment-specific properties, appearing susceptible to viral infection, and completing the innate immune signaling pathways (20). Based on this, the susceptibility to viral infection and intact innate immune signaling of IECs are taken into account in the exploration of immune responses against enterovirus infections in intestine. In this study, we presented that human IECs (FHC and HT29 cells) are susceptible to enterovirus infection to activate the innate immune signaling involved in TLR3/IFN as a compatible model. Although STING-dependent signaling is suppressed in the most cancerous intestine cells (34), we reported that similar to the normal FHC cells, cancerous HT29 cells showed active innate immune response in TLR3/IRF1/IFN signaling upon enterovirus infection.

The current study initially demonstrates that infections by three kinds of enteroviruses (EV71, CVB3, and PV1) lead to the predominant production of type III IFNs (IFN-λ1 and IFN-λ2/3). TLRs initiate innate immune response by the recognition of pathogens and the induction of interferons and proinflammatory cytokines (45). TLRs are essentially expressed in intestine, with TLR3 expressed abundantly in human small intestine and colon (46). TLR3 is induced as an essential receptor to initiate antiviral response by activating the type III IFN in rotavirus-infected human IECs (26, 27). Our results expand the notion of antiviral response mediated by TLR3-induced type III IFN to a range of enteroviruses, including EV71, CVB3, and PV1. As an inducible factor, IRF1 exerts antiviral activity in the early stages of viral replication in the respiratory epithelial cells (19, 31). This study demonstrates that EV71 preferentially promotes the expression and nuclear translocation of IRF1. Notably, IRF1 is required for EV71-induced TLR3 expression, while TLR3 contributes to EV71-activated IRF1 expression, suggesting a positive feedback loop between IRF1 and TLR3 whereby IRF1 also induces TLR3 transcription as previously reported (35). We speculated that activated TLR3 signaling might simultaneously promote IRF1 expression upon EV71 infection. In addition, EV71-induced IFN-λ production determines the induction of IRF1 (17), indicating that there is a dynamic relationship between IRF1 and type III IFN. Collectively, these results exposed that enteroviruses induce predominant production of type III IFNs through activating the TLR3/IRF1 signaling pathway.

We further reveal that type III IFN subsequently induces an intrinsic antiviral action against the enterovirus infection in human and mouse intestines. It is in agreement with a recent study reporting that type III IFN restricts EV71 infection in goblet cells (21). In the innate antiviral defenses, type III IFNs have functions similar to that of type I IFNs, and the signaling cascades involved and the ISGs expressed are comparable between the two types of IFNs (47). Although EV71 predominantly induces type III IFNs in IECs, type I IFN can protect mice against EV71 infection (48). Systemic treatment of suckling mice with IFN-λ represses rotavirus replication in the gut (26). The protective property of type III IFNs against EV71 replication in mice is further documented in this study by the demonstration that IFN-λ2 improves survival of mice after EV71 infection.

Type I and type III IFNs exert antiviral function with distinct kinetics. Type I IFN signaling is characterized by an acute strong upregulation of ISGs, conferring fast antiviral protection but concomitantly promoting inflammation at the site of infection (17). Conversely, the antiviral protection by slow-acting type III IFNs is characterized by a less potent (49) and delayed but more sustained (11) activation of ISGs. Nevertheless, the expression of type III IFN receptor is limited to epithelial cells, and IFN-λ induces robust antiviral protection specifically in IECs (49, 50). The gut epithelium produces a large amount of IFN-λ, but not IFN-α/β, in response to enteroviral infection in mice (51). We reported that type III IFNs suppress EV71 infection in mice, indicating that type III IFNs afford superior protection against enteroviral infection in the intestinal epithelium.

In general, the findings of the current study raise the hypothesis that intestinal epithelial cells act as the first line of defense against enteroviral infection in humans and in mice. During enterovirus infection, the viral dsRNA is recognized by TLR3, leading to triggering of downstream signaling early in the viral replication cycle. Subsequently, IRF1 is translocated to the nucleus, resulting in the differential expression of type III IFNs and activation of innate immune antiviral response in intestinal epithelial cells. In conclusion, the present study reveals a distinct mechanism by which the host elicits immune responses against enterovirus infections in the intestine through activating the TLR3/IRF1/type III IFN signaling pathway.

## MATERIALS AND METHODS

### Human specimens.

Human intestinal tissue samples were acquired from the School of Forensic Medicine, Kunming Medical University. Four specimens were obtained from deceased patients who were confirmed to be infected with EV71, and three specimens were from uninfected subjects and used as negative controls. The tissues were fixed in formalin and embedded in paraffin. The information on these specimens is included in Table 1.

### Animal studies.

C57BL/6J mice were purchased from the Hubei Provincial Center for Disease Control and Prevention. Three-day-old C57BL/6J mice were infected by intraperitoneal injection of EV71, 1 × 10^7^ PFU, suspended in 20 μl PBS. All mice were housed in the specific-pathogen-free (SPF) animal facility at Wuhan University.

### Ethics statement.

The study was conducted according to the principles of the Declaration of Helsinki and approved by the Institutional Review Board (IRB) of the College of Life Sciences, Wuhan University, in accordance with its guidelines for the protection of human subjects.

All animal studies were performed in accordance with the principles described by the Animal Welfare Act and the National Institutes of Health guidelines for the care and use of laboratory animals in biomedical research. All procedures involving mice and experimental protocols were approved by the Institutional Animal Care and Use Committee (IACUC) of the College of Life Sciences, Wuhan University (permit number 19030A).

### Mouse organ isolation.

Mouse tissues from different organs were harvested following the procedure previously reported (52, 53). Briefly, the mouse was euthanized with carbon dioxide and placed on its back with 70% ethanol sprayed. A middle-line incision was made from neck to tail, to expose the peritoneal cavity by opening skin and abdominal walls. Subsequently, all the body organs were carefully pulled over one by one, such as brain, lung, heart, liver, small intestine, and kidneys, and then any remaining fats were discarded on a paper towel or dish containing PBS. Finally, half of each organ was subjected to IHC by fixation in 4% formaldehyde and another half was subjected to qPCR by grinding in PBS with a tissue grinder, following the standard method of RNA extraction.

### Cell cultures.

Human intestinal cells (FHC, HCoEpiC, HT29, HCT116, DLD1, LoVo, and SW48), human rhabdomyosarcoma (RD) cells, and a human embryonic kidney cell line (HEK293T) were purchased from the American Type Culture Collection (ATCC, Manassas, VA, USA). The cells were maintained in Dulbecco’s modified Eagle’s medium (DMEM) (Gibco, Grand Island, NY, USA), supplemented with 10% fetal bovine serum (FBS) (Gibco), 100 U/ml penicillin, and 100 μg/ml streptomycin sulfate. Cells were maintained at 37°C in a 5% CO_2_ incubator.

### Virus infection.

The EV71 virus strain (Xiangyang-Hubei-09) was previously isolated in our laboratory (GenBank accession no. JN230523.1) (33, 54, 55), CVB3 (Nancy strain) was kindly provided by Zhanqiu Yang (Wuhan University, China), and PV1 (vaccine strain) was obtained from the China Center for Type Culture Collection (CCTCC) (Wuhan, China). All viral strains were propagated in RD cells. For viral replication assays, cells were infected with the virus at indicated multiplicities of infection (MOI), and the unbound virus was washed out 2 h later. Subsequently, the infected cells were cultured in fresh serum-free medium for indicated time intervals.

### Reagents.

Recombinant human IFN-α2b was purchased from Sinobioway Medicine (Tianjin, China). Recombinant human IFN-λ1 was purchased from Novoprotein (Shanghai, China). Recombinant murine IFN-λ2 was purchased from PeproTech (Rocky Hill, NJ, USA). Recombinant human IFN-γ (catalog no. 285-IF) was obtained from R&D Systems (Minneapolis, MN, USA). Lipofectamine 2000 was purchased from Invitrogen (Carlsbad, CA, USA). Rabbit anti-EV71 VP1 antibody was obtained from Abnova (Taipei, Taiwan). Rabbit anti-TLR3 and anti-IL-28+IL-29 antibodies were purchased from Abcam (Cambridge, United Kingdom). Rabbit anti-IRF1 antibody was obtained from Cell Signaling Technology (Beverly, MA, USA). Rabbit antibodies to β-actin and EV71 3C were purchased from ABclonal Technology (Wuhan, China). Mouse anti-glyceraldehyde-3-phosphate dehydrogenase (anti-GAPDH) was purchased from Proteintech Group (Rosemont, IL, USA). Mouse monoclonal anti-dsRNA J2 antibody was obtained from Scicons (Budapest, Hungary). Rabbit anti-dsRNA (J2) was purchased from Absolute Antibody (Wilton, United Kingdom). Mouse anti-MUC2, anti-lysozyme C, and anti-ChrA were purchased from Santa Cruz Biotechnology (Santa Cruz, CA, USA). Poly(I·C) was obtained from InvivoGen (San Diego, CA, USA).

### Virus titer determination.

For the determination of 50% tissue culture infectious dose (TCID_50_), RD cells were seeded in 96-well plates and incubated for 2 h in 10-fold serial dilutions of cell culture supernatants. Subsequently, the medium was discarded, the cells were washed with PBS, and cultures were continued for 2 to 3 days in serum-free DMEM. For plaque assay, RD cells were plated in 12-well plates, exposed to EV71 for 2 h, and maintained in DMEM-agarose medium for 2 to 3 days. Then, the cells were fixed with 4% paraformaldehyde for 30 min, washed three times with PBS, and stained with crystal violet dye.

### RNA extraction and quantitative PCR.

Total RNAs from cell or mouse small intestinal tissues were extracted using the TRIzol reagent (Invitrogen) and reverse transcribed into cDNA. Real-time qPCR analysis was performed using the Light Cycler 480 (Roche, Basel, Switzerland) and SYBR green real-time PCR master mix (Bio-Rad, Hercules, CA, USA). The data represent absolute numbers of mRNA copies normalized to GAPDH used as a reference gene. Relative changes in expression were determined by using the comparative threshold cycle (*C_T_*) method. The information on all qPCR primers used in this study is listed in Table 2.

**TABLE 2:** List of primers used for qPCR in this study^*a*^

Primer	Sequence
qRT GAPDH (mouse) F	5′-ATGTTTGTGATGGGTGTGAA-3′
qRT GAPDH (mouse) R	5′-ATGCCAAAGTTGTCATGGAT-3′
qRT IFN-β (mouse) F	5′-GGATCCTCCACGCTGCGTTCC-3′
qRT IFN-β (mouse) R	5′-CCGCCCTGTAGGTGAGGTTGA-3′
qRT IFN-λ2/3 (mouse) F	5′-AGCTGCAGGCCTTCAAAAAG-3′
qRT IFN-λ2/3 (mouse) R	5′-TGGGAGTGAATGTGGCTCAG-3′
qRT TLR3 (mouse) F	5′-ATTCGCCCTCCTCTTGAACA-3′
qRT TLR3 (mouse) R	5′-TCGAGCTGGGTGAGATTTGT-3′
qRT IRF1 (mouse) F	5′-ACTCGAATGCGGATGAGACC-3′
qRT IRF1 (mouse) R	5′-TGCTTTGTATCGGCCTGTGT-3′
qRT GAPDH F	5′-AAGGCTGTGGGCAAGG-3′
qRT GAPDH R	5′-TGGAGGAGTGGGTGTCG-3′
qRT IFN-α F	5′-GATCTCCCTGAGACCCACAG-3′
qRT IFN-α R	5′-GGATCAGCTCATGGAGGACA-3′
qRT IFN-β F	5′-GAGGCTTGAATACTGCCTCAA-3′
qRT IFN-β R	5′-TCCTTGGCCTTCAGGTAATGCAGA-3′
qRT IFN-λ1 F	5′-CTTCCAAGCCCACCACAACT-3′
qRT IFN-λ1 R	5′-GGCCTCCAGGACCTTCAGC-3′
qRT IFN-λ2/3 F	5′-TGCAAGGGGCTGCCACATAG-3′
qRT IFN-λ2/3 R	5′-CTCCAGAACCTTCAGCGTCA-3′
qRT TLR3 F	5′-CCTGGTTTGTTAATTGGATTAACGA-3′
qRT TLR3 R	5′-TGAGGTGGAGTGTTGCAAAGG-3′
qRT IRF1 F	5′-CTGTGCGAGTGTACCGGATG-3′
qRT IRF1 R	5′-ATCCCCACATGACTTCCTCTT-3′
qRT IRF2 F	5′-AGGCAAACAGTACCTCAGCA-3′
qRT IRF2 R	5′-CGGTTTGTTGGAAGTGACGA-3′
qRT IRF3 F	5′-GACCCTCACGACCCACATAA-3′
qRT IRF3 R	5′-CAGAAGTACTGCCTCCACCA-3′
qRT IRF5 F	5′-CCAGGACGGAGATAACACCA-3′
qRT IRF5 R	5′-GGCTCTTGTTAAGGGCACAG-3′
qRT IRF7 F	5′-TACCATCTACCTGGGCTTCG-3′
qRT IRF7 R	5′-CTGCTGCTATCCAGGGAAGA-3′
qRT IRF9 F	5′-GCAACTCAGGATGGCATCAG-3′
qRT IRF9 R	5′-CCTTGAAGAAGGCAGCATCC-3′
qRT IFIT1 F	5′-CCTCCTTGGGTTCGTCTACA-3′
qRT IFIT1 R	5′-GGCTGATATCTGGGTGCCTA-3′
qRT MX1 F	5′-ACACATGCTGAACATCACAGCTT-3′
qRT MX1 R	5′-ACACGGCACTCATGCTCCTAA-3′
qRT PKR F	5′-AAAGCGAACAAGGAGTAAG-3′
qRT PKR R	5′-GATGATGCCATCCCGTAG-3′
qRT OASL1 F	5′-TTCCGTCCATAGGAGCCAC-3′
qRT OASL1 R	5′-AAGCCCTACGAAGAATGTC-3′
qRT VIPERIN F	5′-ATACCTGGGCAAGTTGGTGA-3′
qRT VIPERIN R	5′-CGTCAAAGCTGTCACAGGAG-3′
qRT ISG15 F	5′-CAGCGAACTCATCTTTGCCA-3′
qRT ISG15 R	5′-AGGGACACCTGGAATTCGTT-3′
qRT CXCL10 F	5′-TGAAATTATTCCTGCAAGCCAA-3′
qRT CXCL10 R	5′-GACATCTCTTCTCACCCTTCTTT-3′
qRT SOCS3 F	5′-GCGAGGATCCTGGTGACA-3′
qRT SOCS3 R	5′-CCAGGATGGTTCCCTTCAG-3′
qRT CXCL11 F	5′-AAAGCAGTGAAAGTGGCAG-3′
qRT CXCL11 R	5′-CAGATGCTCTTTTCCAGGAC-3′
qRT IFIT3 F	5′-GAAGAGATCAAAGACCAACCAC-3′
qRT IFIT3 R	5′-CCCATTTCCTCACTACCATCC-3′
qRT IFIT2 F	5′-CATCTCAGAACGCCATTGAC-3′
qRT IFIT2 R	5′-TCCTTCACCTTCCTCTTCAC-3′
qRT IFI30 F	5′-AGAAGTCTGCCACTATGCC-3′
qRT IFI30 R	5′-TCACTTGAAGCAAACACTCC-3′
qRT TDRD7 F	5′-GCTAAACTTCCATTGCCCAC-3′
qRT TDRD7 R	5′-TGCCCACATACCTGATAACC-3′
qRT CVB3 F	5′-CGGTACCTTTGTGCGCCTGTT-3′
qRT CVB3 R	5′-GCGGTGCTCATCGACCTGA-3′
qRT PV1 F	5′-CCGTATTGAGCCAGTATGTTTGT-3′
qRT PV1 R	5′-TAGCGAGTAGGTGGAGGTGTTCT-3′
qRT EV71 VP1 F	5′-CTGTGCGAATTAAGGACAG-3′
qRT EV71 VP1 R	5′-GAGTTCCATAGGTGACAGC-3′
qRT EV71 5′UTR F	5′-ACATGGTGCGAAGAGTCTATTGAGCT-3′
qRT EV71 5′UTR R	5′-ACCCAAAGTAGTCGGTTCCGC-3′
qRT EV71 5′UTR probe	5′-TCCGGCCCCTGAATGCGGCTAAT-3′

a The sequences of primers involved in this study. F, forward; R, reverse; qRT, the primers used in quantitative real-time PCR. The mouse genes are indicated.

### Western blot analysis.

Whole-cell lysates of cells were prepared with RIPA buffer (50 mM Tris-HCl, 150 mM NaCl, 0.25% sodium deoxycholate, 1% NP-40, pH 7.4). Protein concentration was determined by Bradford assay (Bio-Rad). The proteins were separated by 12% SDS-PAGE and transferred onto a polyvinylidene difluoride (PVDF) membrane (Millipore, Burlington, MA, USA). The membranes were blocked with 5% skim milk diluted in PBST (PBS with 0.1% Tween) and incubated with the primary antibodies. The bands were detected using a luminescent image analyzer (Fujifilm LAS-4000; Fuji Film, Tokyo, Japan).

### ELISA.

Human IFN-β and IFN-λ1 proteins in the cell culture supernatants were determined using enzyme-linked immunosorbent assay (ELISA) kits purchased from the 4A Biotech Co., Ltd., according to the manufacturer’s instructions.

### shRNA constructions for lentivirus package.

The target sequences of shRNAs for human *TLR3* and *IRF1* genes and the sequence of the negative-control shRNA were as follows: shTLR3-1#, 5′-GCTTGGATGTAGGATTTAACA-3′; shTLR3-2#, 5′-GGTAACGATTCCTTTGCTTGG-3′; shTLR3-3#, 5′-GGAGCACCTTAACATGGAAGA-3′; shIRF1-1#, 5′-GCCAGATATCGAGGAGGTGAA-3′; shIRF1-2#, 5′-GGGATATTGGGCTGAGTCTAC-3′; shIRF1-3#, 5′-GCGTGTCTTCACAGATCTGAA-3′; shNC, 5′-CAACAAGATGAAGAGCACCAA-3′.

HEK293T cells were transfected with the pLKO.1 vector encoding the negative-control or gene-targeting shRNAs, psPAX2, and pMD2.G at the ratio of 4:3:1 using Lipofectamine 2000. Forty-eight hours later, the lentivirus from supernatants was collected to infect FHC or HT29 cells. After an additional 48 h of culture, cells were selected by 1.5 μg/ml puromycin (Sigma-Aldrich, St. Louis, MO, USA). The efficiency of shRNAs to knock down the target genes was evaluated by immunoblot analysis.

### Confocal microscopy.

Cells were seeded on 20-mm coverslips and treated with EV71 for different time intervals and then washed with PBS, fixed with 4% formaldehyde for 30 min, blocked, and permeabilized using saponin buffer (0.1% saponin and 1% BSA in PBS) for 30 min at room temperature. Next, the cells were then incubated with specific primary antibodies overnight at 4°C and with fluorescein isothiocyanate (FITC)-conjugated goat anti-rabbit IgG and Cy3-conjugated goat anti-mouse IgG (Proteintech Group) for 1 h at room temperature. Nuclei were stained with 4′,6-diamidino-2-phenylindole (DAPI) (Roche), 1-μg/ml methanol solution, for 10 min at room temperature. After the last wash with PBS, the stained cells were analyzed by confocal laser scanning microscopy (FluoView FV1000; Olympus, Tokyo, Japan).

### Histological evaluation.

For histological evaluation, sections were stained with hematoxylin and eosin (H&E) and examined using light microscopy. The damage to the small intestine epithelium was assessed blindly as described previously (56). Briefly, the damage was scored as follows: 0 = normal; 1 = hyperproliferation, irregular crypts, and goblet cell loss; 2 = mild to moderate crypt loss (10 to 50%); 3 = severe crypt loss (50 to 90%); 4 = complete crypt loss, surface epithelium intact; 5 = small to medium-sized ulcer (<10 crypt widths); and 6 = large ulcer (>10 crypt widths). Infiltration with inflammatory cells was scored separately for mucosa (0 = normal, 1 = mild, 2 = intermediate, and 3 = severe), submucosa (0 = normal, 1 = mild, 2 = intermediate, and 3 = severe), and muscle and serosa (0 = normal and 2 = moderate to severe). The scores for epithelial damage and inflammatory cell infiltration were added, yielding an overall score ranging from 0 to 12.

### Immunohistochemical analysis.

Mouse tissue samples were fixed in paraformaldehyde and embedded in paraffin. The slides were counterstained with hematoxylin for nuclei and primary antibodies for target proteins. The relative intensity of specific staining was assessed semiquantitatively by Image J software and defined as negative (1+), weakly positive (2+), positive (3+), and strongly positive (4+).

### Statistical analysis.

All experiments were reproductive and repeated at least three times. The results are presented as means ± SD unless stated otherwise. Statistical significance for comparison of two means was determined by unpaired Student’s *t* test. Survival curves were plotted using the Kaplan-Meier method, and significant differences in mouse survival were calculated *via* the log rank test. Statistical significance in mouse weight was calculated by two-way analysis of variance (ANOVA). Statistical significance in Pearson’s correlation analysis was determined by two-tailed *t* test. Analyses were performed using GraphPad Prism 7 software (GraphPad Software, Inc., San Diego, CA, USA). *P < *0.05 was considered statistically significant.
