# Supplementary figures and images for: The TLR3/IRF1/Type III IFN Axis Facilitates Antiviral Responses against Enterovirus Infections in the Intestine
Source: mBio. 2020 Nov 17;11(6):e02540-20. doi: 10.1128/mBio.02540-20 (PMC7683398; doi:10.1128/mBio.02540-20)

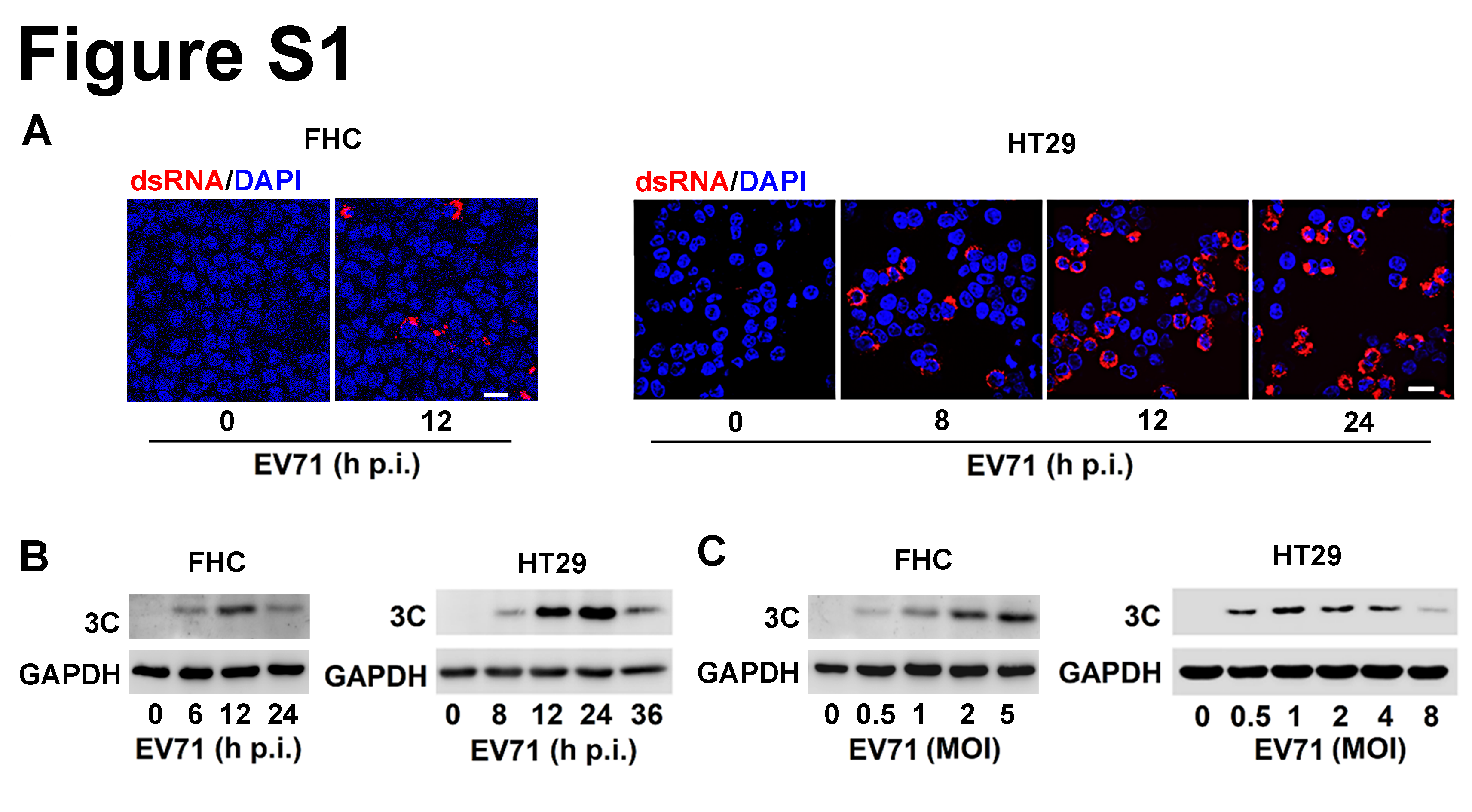

Supplement: FIG S1 [file mBio.02540-20-sf001.tif]

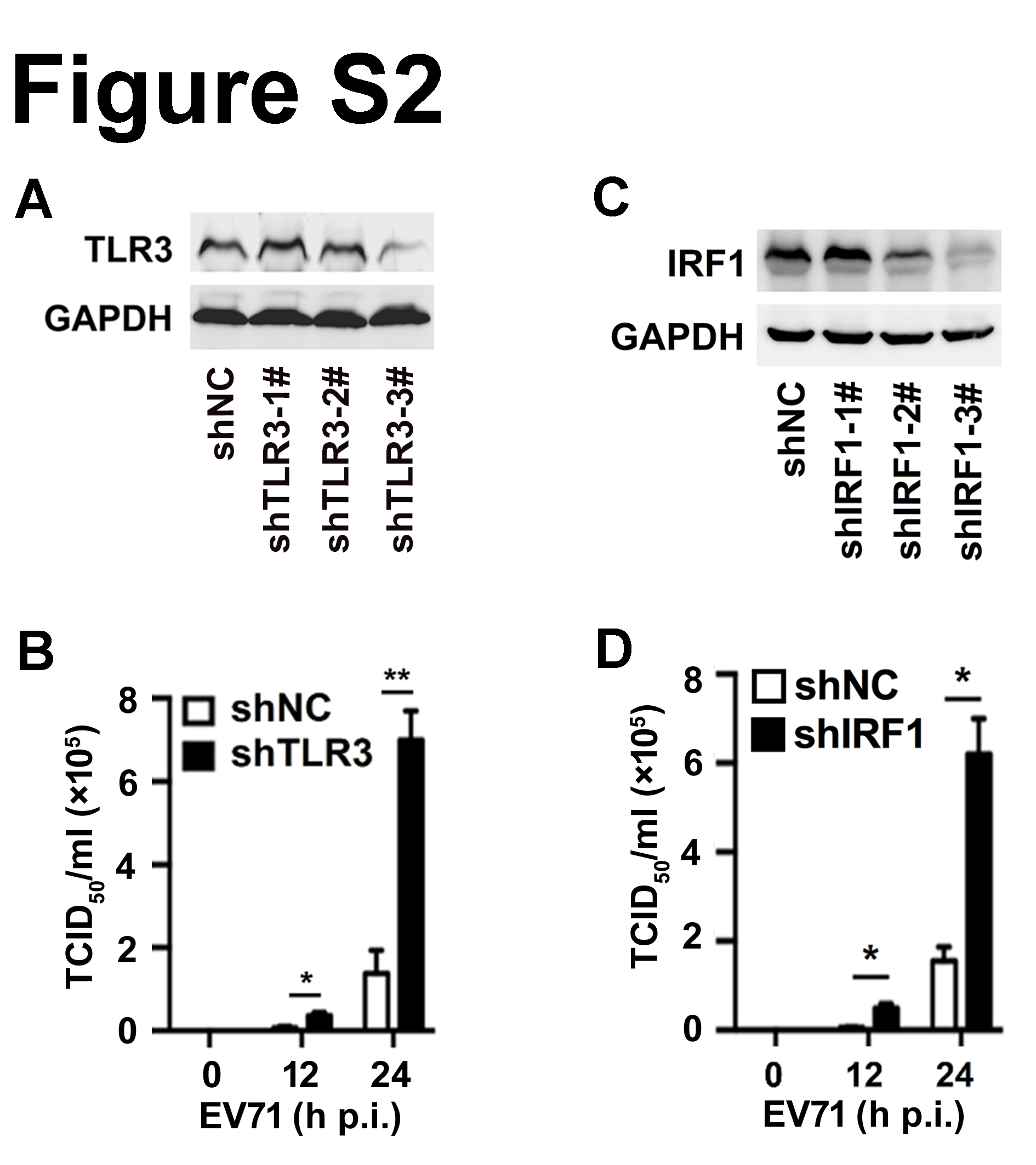

Supplement: FIG S2 [file mBio.02540-20-sf002.tif]

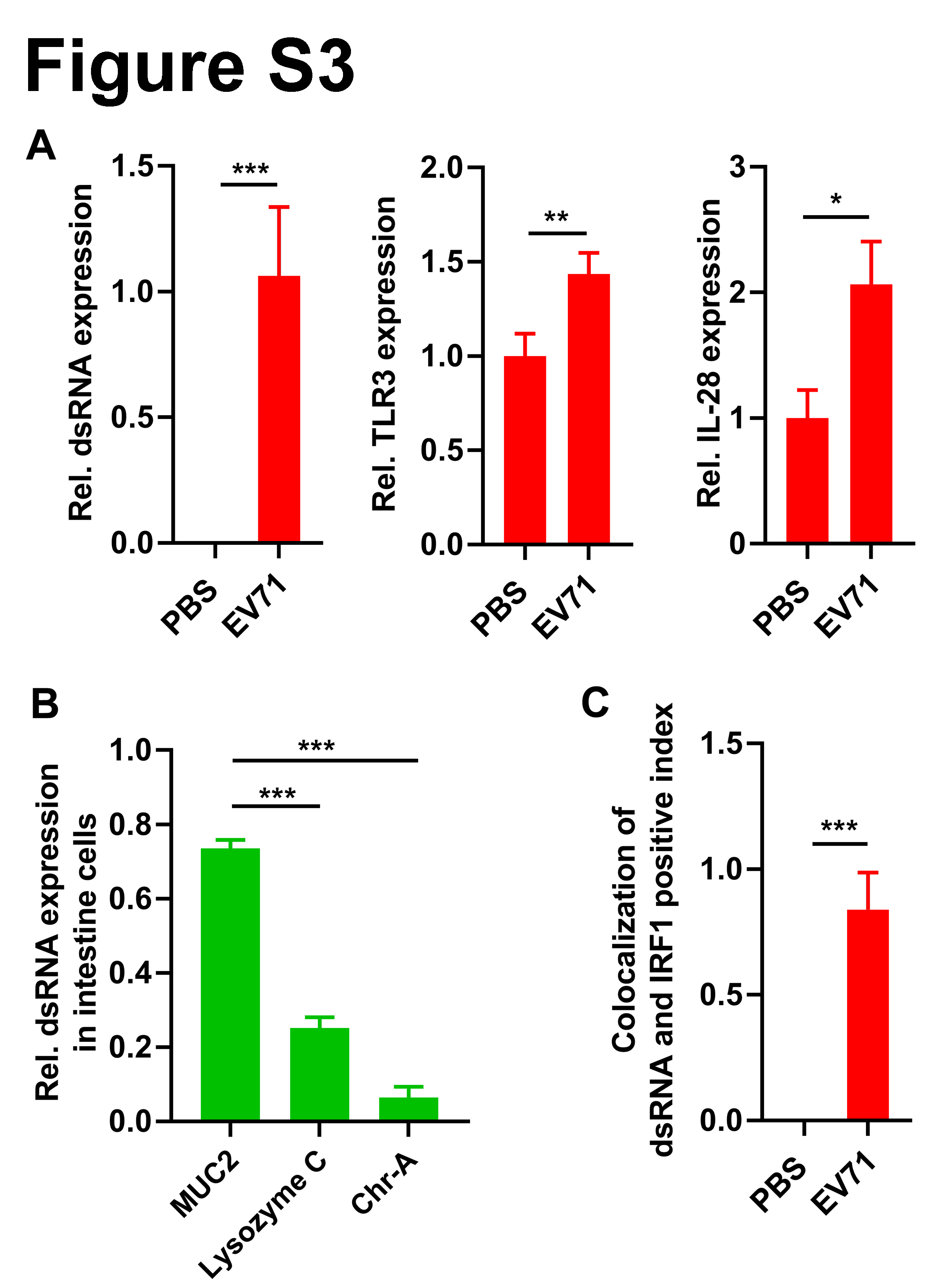

Supplement: FIG S3 [file mBio.02540-20-sf003.tif]
